# The Association of Early Electrocardiographic Abnormalities With Brain Injury Severity and Outcome in Severe Traumatic Brain Injury

**DOI:** 10.3389/fneur.2020.597737

**Published:** 2021-01-08

**Authors:** Jelmer-Joost Lenstra, Lidija Kuznecova-Keppel Hesselink, Sacha la Bastide-van Gemert, Bram Jacobs, Maarten Willem Nicolaas Nijsten, Iwan Cornelis Clemens van der Horst, Joukje van der Naalt

**Affiliations:** ^1^Department of Neurology, University of Groningen, University Medical Center Groningen, Groningen, Netherlands; ^2^Department of Critical Care, University of Groningen, University Medical Center Groningen, Groningen, Netherlands; ^3^Department of Epidemiology, University of Groningen, University Medical Center Groningen, Groningen, Netherlands

**Keywords:** severe traumatic brain injury, cardiac dysfunction, electrocardiography (ECG), computerized tomography (CT), therapy intensity level (TIL), mortality outcome

## Abstract

The aim of this study was to evaluate the frequency of electrocardiographic (ECG) abnormalities in the acute phase of severe traumatic brain injury (TBI) and the association with brain injury severity and outcome. In contrast to neurovascular diseases, sparse information is available on this issue. Data of adult patients with severe TBI admitted to the Intensive Care Unit (ICU) for intracranial pressure monitoring of a level-1 trauma center from 2002 till 2018 were analyzed. Patients with a cardiac history were excluded. An ECG recording was obtained within 24 h after ICU admission. Admission brain computerized tomography (CT)-scans were categorized by Marshall-criteria (diffuse vs. mass lesions) and for location of traumatic lesions. CT-characteristics and maximum Therapy Intensity Level (TILmax) were used as indicators for brain injury severity. We analyzed data of 198 patients, mean (SD) age of 40 ± 19 years, median GCS score 3 [interquartile range (IQR) 3–6], and 105 patients (53%) had thoracic injury. In-hospital mortality was 30%, with sudden death by cardiac arrest in four patients. The incidence of ECG abnormalities was 88% comprising ventricular repolarization disorders (57%) mostly with ST-segment abnormalities, conduction disorders (45%) mostly with QTc-prolongation, and arrhythmias (38%) mostly of supraventricular origin. More cardiac arrhythmias were observed with increased grading of diffuse brain injury (*p* = 0.042) or in patients treated with hyperosmolar therapy (TILmax) (65%, *p* = 0.022). No association was found between ECG abnormalities and location of brain lesions nor with thoracic injury. Multivariate analysis with baseline outcome predictors showed that cardiac arrhythmias were not independently associated with in-hospital mortality (*p* = 0.097). Only hypotension (*p* = 0.029) and diffuse brain injury (*p* = 0.017) were associated with in-hospital mortality. In conclusion, a high incidence of ECG abnormalities was observed in patients with severe TBI in the acute phase after injury. No association between ECG abnormalities and location of brain lesions or presence of thoracic injury was present. Cardiac arrhythmias were indicative for brain injury severity but not independently associated with in-hospital mortality. Therefore, our findings likely suggest that ECG abnormalities should be considered as cardiac mimicry representing the secondary effect of traumatic brain injury allowing for a more rationale use of neuroprotective measures.

## Introduction

Traumatic brain injury (TBI) is an important cause of mortality and morbidity in adults (1). Patients with severe TBI are initially stabilized at the Emergency Department (ED) and admitted to the Intensive Care Unit (ICU) for treatment based on intracranial pressure (ICP)-monitoring to prevent secondary deterioration of brain injury and optimize conditions for brain recovery (2–4). Vital functions are well-known predictors of outcome (5) and are monitored with several tools, including electrocardiography (ECG).

In various neurological and neurosurgical conditions ECG abnormalities are described caused by an elevated sympathetic tone with excessive catecholamine release which can lead to ECG changes that suggest primary myocardial dysfunction and ischemia (6). These cardiac mimicry have been reported in patients with subarachnoid hemorrhage as transient ST-segment changes (7, 8) based on left ventricular wall motion disturbances (9). In patients with ischemic or hemorrhagic stroke, ECG abnormalities with arrhythmias are frequently observed and associated with 3-month mortality (10), especially when lesions are located in the right-sided insular cortex (11).

Despite the growing literature on the peripheral interactions of TBI (12), only small case series or single cohort studies of patients with isolated TBI have highlighted the link between TBI and cardiac dysfunction, without data on early ECG abnormalities and outcome. The frequency of ECG abnormalities is related to Glasgow Coma Scale (GCS) on admission (13), but not to brain severity indices like computerized tomography (CT)-characteristics or ICP-treatment (14, 15) ECG abnormalities in patients with TBI can be regarded as a secondary effect of brain injury or an expression of primary cardiac dysfunction, due to thoracic injury (16, 17) and for that reason more difficult to interpret than in neurovascular diseases.

Therefore, we aimed in this current study to evaluate the incidence and clinical significance of ECG abnormalities in patients with severe TBI with and without thoracic injury and their association with brain injury severity and outcome. First, we determined the incidence of ECG abnormalities among patients with severe TBI admitted to the ICU by evaluating early ECG recordings obtained within 24 h after ICU admission. This time window was chosen because existing literature suggests that cardiac dysfunction occurs early after brain injury (18). Second, we evaluated the relationship of ECG abnormalities with brain injury severity defined by CT-characteristics and Therapy Intensity Level (TIL). Lastly we assessed the predictive value of these ECG abnormalities on in-hospital mortality.

## Materials and Methods

In this retrospective study data of patients aged ≥16 years with severe TBI (GCS score <8 after initial stabilization) with ICP-monitoring admitted to the ICU of the University Medical Center Groningen (UMCG) from 2002 till 2018 were analyzed. Data were collected from medical records and ambulance forms. Patients with ECG recordings obtained within 24 h after ICU admission were included. Patients with pre-existing cardiac disease like myocardial infarction, (paroxysmal) arrhythmias, congenital heart disease (cardiomyopathy), or the presence of a pacemaker or implantable cardioverter defibrillator were excluded for analyses. GCS score and pupillary reactivity were documented on admission including hypoxia (oxygen saturation <90%), hypotension (systolic blood pressure <90 mm Hg or requiring vasopressors) within the first 24 h that are known as predictors of outcome (5). Trauma severity was defined according to the Injury Severity Score (ISS) (19) derived from the Abbreviated Injury Scale (AIS) (20). Thoracic injury was defined as an AIS-Thorax score of ≥1, such as the presence of at least one rib fracture or a contusion of the sternum. Laboratory measurements of potassium [hypokalemia (<3.5 mmol/l) and hyperkalemia (>5.0 mmol/l)] were frequently determined on a Radiometer ABL 700/800 series analyzer and with standard ICU equipment. A waiver for this study was obtained by the Medical Ethical committee of the UMCG (M11.096947).

### Computerized Tomography

All patients underwent a brain CT-scan directly after stabilization at the ED or during ICU admission in case of secondary deterioration. CT-scans were evaluated by a board-certified radiologist and brain injury severity was categorized according to the Marshall-criteria based on the absence or presence of diffuse injury (grade 1–4) or evacuated or non-evacuated mass lesion >25 cc (grade 5–6) (21). The highest Marshall-score obtained within 24 h of ICU admission was used for analysis. Location of traumatic lesions (subarachnoid hemorrhage, contusion, extra- or intradural hematoma, petechial hemorrhages) were recorded separately for both hemispheres per region (frontal, temporal, parietal, occipital, brainstem).

### Intracranial Pressure Treatment

Patients with severe TBI were treated in accordance with international guideline-based management with ICP-monitoring (RAUMEDIC® NPS-2). The treatment protocol aims to maintain ICP ≤ 20–25 mm Hg and cerebral perfusion pressure (CPP) between 60–70 mm Hg with stepwise escalation of therapy according to Therapy Intensity Level (TIL) including sedation, cerebrospinal fluid (CSF) drainage, hyperosmolar therapy [mannitol and/or hypertonic saline (HTS)], and second-tier therapy (surgical evacuation and/or barbiturates) (2, 3). Every hour ICP, CPP and TIL score were noted. For the current study the maximum TIL (TILmax) within 24 h of ICU admission was used for analysis.

### Electrocardiographic Recording

The first abnormal ECG obtained at the ED or within 24 h of ICU admission was used for analysis. A 12-lead ECG was obtained and recorded with CardioPerfect equipment (Cardio Control) and digitally stored in MUSE^TM^ (General Electric Company) or manually in the patient record. Digitally stored ECGs were automatically examined for rate (abnormal: <60 or >100 beats/min) and intervals [abnormal: *PR*: <120 ms, >210 ms, *QRS*: >120 ms, *QTc* (22): >450 ms in men and >460 ms in women]. The presence of ventricular repolarization disorders (ST-segment, T-wave, presence of pathological Q-wave or U-wave), conduction defects, and cardiac arrhythmias (supra-/ventricular) were independently scored by two cardiologist-intensivists (IH and LK-K) according to standardized criteria (23). We considered an ECG as abnormal with presence of the following characteristics: A. ventricular repolarization disorder and/or B. conduction disorder and/or C. cardiac arrhythmia.

### Statistical Analysis

Analysis is performed with IBM SPSS 23.0 and software package R. All normally distributed variables are presented as means, standard deviations and 95% confidence intervals and mean values between groups are analyzed with independent-samples *t*-tests. Non-normally continuous data are presented as medians and ranges and comparison between groups with the Mann-Whitney test. All nominal or categorical variables are described as frequencies and percentages. Comparison between groups was performed using Fisher's exact test. A *p*-value of <0.05 was considered to be statistically significant.

With logistic regression analysis we determined the predictive value of several established factors for outcome on presence of ECG abnormalities. As second step the predictive value of ECG abnormalities for in-hospital mortality was determined. First, univariate analysis was done with the following factors: baseline predictors derived from the literature (5) [age, hypoxia, hypotension, ISS, and brain injury severity (GCS, pupillary reactivity presence of diffuse injury)] combined with location of isolated traumatic brain lesions, increase of TIL, and electrolyte disorders (potassium). Second, variables with a *p*-value of <0.10 in univariate regression were used in multivariate logistic regression. Associations between independent- and depend variables were displayed as odds ratios (OR) and the discriminatory power as Nagelkerke's *R*^2^.

## Results

From a total of 298 consecutive patients with severe TBI who received ICP-monitoring, 16 patients were excluded due to history of cardiac disease and 84 patients due to absence of ECG within 24 h after ICU admission. Clinical data of 198 patients with ECG recordings were available for analysis (Table 1), with similar patient characteristics in comparison to excluded patients. Patients had a mean (SD) age of 40 ± 19 years and median (IQR) admission GCS score of 3 (3–6), 78% were male. Two third of injuries were road traffic accidents (67%). Median ISS (IQR) was 29 (23–34) with concomitant thoracic injury (AIS-Thorax) in 105 patients (53%). Mean potassium levels were abnormal in 20% of the patients with respectively 16.5% hypokalemia and 3.5% hyperkalemia. Hypoxia was present in 12% and hypotension in 16% of patients until 24 h after ICU admission.

**Table 1:** Patient characteristics divided by in-hospital mortality.

**Patient characteristics**	**Study cohort *n* = 198**	**Survivors *n* = 139 (70%)**	**Non-survivors *n* = 59 (30%)**	***P*-value**
Mean age at injury (SD)	40 years (19)	37 years (18)	46 years (19)	0.004
Male	154 (78%)	78%	76%	0.852
Place of accident				0.340
Road traffic	132 (67%)	68%	63%	
Home	32 (16%)	14%	22%	
Public space	16 (8%)	8%	8%	
Work	12 (6%)	6%	7%	
Sports	6 (3%)	4%	0%	
Mechanism of injury				0.483
Collision	110 (56%)	58%	49%	
Fall	71 (36%)	35%	39%	
Other	17 (9%)	7%	12%	
Hypoxia	24 (12%)	8%	22%	0.008
Hypotension	31 (16%)	12%	25%	0.019
ISS	29 (24–35)	27 (22–35)	29 (25–38)	0.067
AIS-Head	5 (4–5)	4 (4–5)	5 (5–5)	<0.001
AIS-Thorax	105 (53%) 3 (2–4)	79 (57%) 3 (2–4)	26 (44%) 3 (2–4)	0.031
GCS^*^	192 (97%) 3 (3–6)	3 (3–6)	3 (3–6)	0.330
Pupil reactivity^*^	180 (91%)			0.021
Bilateral	131 (73%)	77%	64%	
Unilateral	21 (12%)	13%	9%	
Absent	28 (16%)	10%	27%	
CT-scan (Marshall-criteria)				<0.001
Diffuse injury II	77 (39%)	50%	14%	
Diffuse injury III	46 (23%)	22%	25%	
Diffuse injury IV	13 (7%)	4%	12%	
Mass lesion V	11 (5%)	7%	3%	
Mass lesion VI	51 (26%)	17%	46%	

*Values are represented as medians (interquartile range) or numbers (%), unless indicated differently*.

* *on admission. AIS, abbreviated injury scale; CT, computerized tomography; GCS, glasgow coma scale; ISS, injury severity score; SD, standard deviation; TBI, traumatic brain injury*.

### Incidence of Electrocardiographic Abnormalities

Patients showed in 88% abnormalities consisting of A. ventricular repolarization disorders (57%), B. conduction disorders (45%), and C. cardiac arrhythmias (38%).

Specific ECG characteristics were observed as follows:

*A. Ventricular repolarization disorders:* most common were ST-segment abnormalities (36%). An abnormal T-wave occurred in 28%. Presence of pathological Q-wave or U-wave was seldom, both in 1% of patients.

*B. Conduction disorders:* QTc-prolongation (36%) was the most common conduction disorder and significantly more present in men (*p* = 0.023) (40 vs. 22%). An abnormal QRS-interval was present in 11% and an abnormal PR-interval in 5% of patients.

*C. Cardiac arrhythmias:* were mostly supraventricular origin (95%) with respectively 66% sinoatrial node (sinus tachycardia 35% and sinus bradycardia 31%) and 29% atrial or atrioventricular (AV) junctional dysfunction. Ventricular arrhythmias were present in 5% of patients.

No further differences between men and women were observed for other ECG characteristics.

### Computerized Tomography Characteristics

According to the Marshall-criteria, diffuse brain injury (grade 1–4) was present in 69% and an mass lesion (grade 5–6) was present in 31% of patients. Frequency of ventricular repolarization disorders was not significantly different between patients with diffuse injury or mass lesion (54 vs. 65%, *p* = 0.166). Conduction disorders were significantly more present in patients with diffuse injury compared to patients with mass lesions (respectively 50 vs. 34%, *p* = 0.045). Although the incidence of cardiac arrhythmias was comparable in patients with diffuse injury or mass lesion, namely 38 and 37% (Table 2), the increase in grading of diffuse brain injury was associated with more arrhythmias (*p* = 0.042). If traumatic lesions were present, ECG abnormalities were observed in 87–93% of patients, depending on the type of traumatic lesion. Subarachnoid hemorrhage (64%) and hemorrhagic contusion (63%) were most common, with ventricular repolarization disorder in 57–56%, conduction disorders in 39–44% and cardiac arrhythmias in 39–34% of patients (respectively Table 3).

**Table 2:** Incidence of electrocardiographic abnormalities divided by Marshall-criteria.

**Marshall-criteria**	**Study cohort** ***n*** **=** **198**				**Abnormal ECG *n* = 175 (88%)**	**Ventricular repolarization disorder *n* = 113 (57%)**	**Conduction disorder *n* = 89 (45%)**	**Cardiac arrhythmia *n* = 75 (38%)**
		***n***	**%**	**ns%**	**%**	**%**	**%**	**%**
Diffuse injury	II	77	39	10	88	51	46	30
	III	46	23	33	91	61	54	46
	IV	13	7	54	85	46	62	62
Mass lesion	V	11	5	18	73	64	18	36
	VI	51	26	53	90	65	37	37

*Values are represented as numbers or percentages. ECG, electrocardiography; ns, non-survivors*.

**Table 3:** Incidence of electrocardiographic abnormalities divided by type and location of traumatic lesions.

**Traumatic lesions**	**Study cohort** ***n*** **=** **198**		**Abnormal ECG *n* = 175 (88%)**	**Ventricular repolarization disorder *n* = 113 (57%)**	**Conduction disorder *n* = 89 (45%)**	**Cardiac arrhythmia *n* = 75 (38%)**
	***n***	**%**	**%**	**%**	**%**	**%**
Subarachnoid hemorrhage	127	64	87	57	39	39
Basal	9	7	78	11	67	56
Cortical	88	69	85	59	39	35
Multiple	30	24	97	63	33	47
Hemorrhagic contusion	124	63	88	56	44	34
L temporal (+)	37	24	89	51	41	35
L temporal (-)	14	8	86	50	43	21
R temporal (+)	36	23	78	61	22	28
R temporal (-)	16	10	69	50	13	31
Subdural hematoma	89	45	90	55	44	45
L (-)	33	37	91	55	46	42
R (-)	31	35	87	52	42	39
Petechial hemorrhage	41	21	93	49	54	29
Brainstem	15	37	93	53	47	40
Thalamus/IC	16	39	94	50	50	31
Corpus callosum	10	24	100	60	60	60
Epidural hematoma	32	16	88	59	47	34
L (-)	14	44	78	71	36	21
R (-)	15	47	93	47	47	47

*Values are represented as numbers or percentages. ECG, electrocardiography; IC, Internal capsule; L, left-sided; R, right-sided*.

*For the concerning type of traumatic lesion: (-) isolated, (+) non-isolated*.

### Therapy Intensity Level

Increase of TIL within the first day of ICU admission occurred in 39% of patients, with the following TILmax levels: CSF drainage (15%), hyperosmolar therapy (13%; 65% both mannitol and HTS, 22% only mannitol, 13% only HTS), surgical evacuation (11%) and barbiturate therapy (1%). ECG abnormalities varied from 79–92% without significant differences between TIL levels (*p* = 0.280). Ventricular repolarization disorders were present in 50–66% of patients, without differences between TIL levels (*p* = 0.658). Conduction disorders were observed in 38–50% of patients without significant differences between TIL levels (*p* = 0.914). Only in patients treated with hyperosmolar therapies, cardiac arrhythmias were significantly more frequent (65%, *p* = 0.022) compared to other TIL levels (sedation 35%, CSF-drainage 35%, surgical evacuation 24%). Hypotension until the first day of ICU admission was observed in 23% of patients (*n* = 6/26) treated with hyperosmolar therapies without an association with cardiac arrhythmias (*p* = 0.628).

### Prediction of ECG Abnormalities and In-hospital Mortality

In-hospital mortality was 30%, with brain injury in 92% as primary cause of death and in 8% accompanied by systemic complications of which four patients died by cardiac arrest.

Univariate regression analysis showed no association between ECG abnormalities with left- or right isolated located traumatic lesions, thoracic injury, electrolyte disorders, or outcome predictors. Regarding specific EGC characteristics, multivariate analysis showed that only hypoxia (odds ratio (OR) = 3.88; confidence interval (CI) 1.20–12.54; *p* = 0.024; *R*^2^ = 0.151) was independently associated with presence of conduction disorders. Cardiac arrhythmias were associated with a higher ISS (*p* = 0.045).

Non-survivors had a significantly higher age (*p* = 0.005), more hypoxia (*p* = 0.007) or hypotension (*p* = 0.016), more arrhythmias (*p* = 0.015), more presence of diffuse brain injury (*p* = 0.001), and more frequently an increase of TIL (*p* < 0.001) (Table 4). In multivariate regression analysis in-hospital mortality was independently associated with hypotension (OR = 4.13; CI 1.16–14.78; *p* = 0.029; *R*^2^ = 0.271) and presence of diffuse brain injury (OR = 0.33; CI 0.14–0.82; *p* = 0.017; *R*^2^ = 0.271) (adjusted for age, hypoxia, cardiac arrhythmias, ISS, pupil reactivity, and increase of TIL).

**Table 4:** Univariate- and multivariate linear regression analysis.

**Predictor**	**Univariate OR (95% CI)**	***P*-value**	**Multivariate OR (95% CI)**	***P*-value**
**ECG abnormalities**				
Age	1.01 (0.98–1.03)	0.486	..	..
Hypoxia	1.51 (0.33–6.89)	0.595	..	..
Hypotension	1.27 (0.35–4.56)	0.714	..	..
Thoracic injury	0.48 (0.19–1.20)	0.114	..	..
ISS	1.05 (0.10–1.10)	0.075	..	..
GCS	0.84 (0.67–1.05)	0.127	..	..
Bilateral pupil reactivity	0.82 (0.28–2.40)	0.709	..	..
Diffuse brain injury	1.20 (0.48–2.99)	0.703	..	..
Isolated left sided lesion	0.77 (0.28–2.12)	0.610	..	..
Isolated right sided lesion	0.71 (0.24–2.09)	0.534	..	..
Increase of TIL	0.56 (0.23–1.33)	0.186	..	..
Potassium abnormalities	1.33 (0.37–4.88)	0.664	..	..
**In-hospital mortality**				
Age	1.02 (1.01–1.04)	0.005	1.02 (1.0–1.04)	0.106
Hypoxia	3.29 (1.38–7.86)	0.007	2.05 (0.58–7.32)	0.267
Hypotension	2.62 (1.20–5.74)	0.016	4.13 (1.16–14.78)	0.029
ECG abnormalities	2.18 (0.71–6.70)	0.175	..	..
A. Ventr. rep. dis.	1.39 (0.75–2.60)	0.297	..	..
B. Conduction dis.	0.95 (0.52–1.76)	0.871	..	..
C. Cardiac arrhythmias	2.16 (1.16–4.03)	0.015	1.96 (0.88–4.34)	0.097
ISS	1.03 (1.00–1.06)	0.072	1.00 (0.96–1.04)	0.934
GCS	0.92 (0.77–1.01)	0.328	..	..
Bilateral pupil reactivity	0.53 (0.27–1.05)	0.070	1.01 (0.40–2.58)	0.980
Diffuse brain injury	0.32 (0.17–0.61)	0.001	0.33 (0.14–0.82)	0.017
Increase of TIL	3.62 (1.91–6.83)	<0.001	2.09 (0.93–4.73)	0.076

*CI, confidence interval; CSF, cerebrospinal fluid; CT, computerized tomography; ECG, electrocardiography; GCS, glasgow coma scale; OR, odds ratio; ISS, injury severity score; TIL, therapy intensity level*.

## Discussion

Within a large sample of patients we observed that early ECG abnormalities are common in patients with severe TBI and are associated with brain injury severity but not to in-hospital mortality. Increased grading of diffuse brain injury and Therapy Intensity Level as indicators of brain injury severity were associated with more cardiac arrhythmias. ECG abnormalities were not associated with location of traumatic brain lesions nor with the presence of thoracic injury. These findings lend further support to the assumption that ECG abnormalities might be regarded as secondary effect of brain injury severity in the early phase after injury and may allow a more rational approach regarding neuroprotective measures.

In our cohort of patients with severe TBI, ECG abnormalities were very often observed in the acute phase after injury, in 88% of patients. Ventricular repolarization disorders were present in more than half of the patients, mostly comprising ST-abnormalities. Conduction disorders occurred in 45%, including mostly QTc-prolongation and arrhythmias, mostly of supraventricular origin, were present in 38% of patients. We observed more ECG abnormalities in comparison to findings of earlier studies, which might be related to their restricted age inclusion criteria (up to 50 years) and the presence of isolated TBI (24) or less severe head injured patients (13). These frequencies are also much higher compared to baseline ECG characteristics of the healthy adult patients from the LifeLines Cohort from our hospital with only 7.4% cardiac arrhythmias and 1.5% QTc-prolongation, with the latter being more prevalent in men in line with findings from our study (25). This cohort could be considered as a reliable base line comparison to our patients, and therefore suggests a secondary effect of the injury itself.

However, despite this high frequency of ECG abnormalities after injury, it is unclear whether these have to be interpreted as a secondary effect of brain injury or as primary cardiac dysfunction. First, it has to be determined if these ECG abnormalities are related to direct injury to a specific location within the brain or reflect the extent of diffuse injury of the traumatic cerebral insult (18). Cardiac mimicry are described in both neurological and neurosurgical patients (6). In patients with stroke, involvement of the insular cortex has been associated with QTc-prolongation (26) and ECG abnormalities occurred more frequently when lesions were located in the right-sided insular cortex (11). It has been demonstrated that stimulation of the left insular cortex causes parasympathetic cardiac responses with the right insular cortex showing sympathetic responses (27). Subsequently, arrhythmias and fatal cardiac outcome have been related to intracerebral infarct location, and a role of the insula in the occurrence of arrhythmia has been confirmed in later studies in ischemic stroke (28–31). However, in our cohort of patients with severe TBI, we did not find an association between isolated left- or right located temporal traumatic lesions and ECG abnormalities. An explanation for this absent relation of ECG abnormalities with a specific region in patients with severe TBI, could be that in stroke more circumscribed areas of injured brain are present while in TBI the more global effects of blunt head injury might obscure the relationship between the insular region and arrhythmias. This assumption is confirmed by our observation that arrhythmias were more frequent with increasing grading of diffuse brain injury. This finding is in line with the postulation that more severely injured patients have a higher sympathetic activation as a result of the stress response to the trauma itself (32). Diffuse brain injury is likely a surrogate for a greater degree and extent of diffuse energy transition during the accident.

The maximum TIL was used as another indicator for brain injury severity since this is regarded a reliable determinant of ICP management in patients with TBI (3). In our patients with severe TBI, ECG abnormalities with ventricular repolarization disorders and conduction defects were often observed and not associated with different maximum TIL levels. However, only arrhythmias were significantly more frequently observed in patients who required ICP escalation hyperosmolar therapies, without an association with well-known adverse events like electrolyte disorders and acute hypotension in the acute phase of injury (33). These results are in line with observations of raised ICP with reversible cardiac arrhythmias in animal studies (34) and in a small human case series of patients with varying intracranial conditions (35). Arrhythmias were present in one out of three patients and associated with in-hospital mortality. These findings are in line with cardiac dysfunction in patients with (isolated) TBI (13, 24) and even higher than frequently observed ECG abnormalities in patients with ischemic (60%) or hemorrhagic stroke (50%) during admission, in which arrhythmias are associated with 3-month mortality (10).

However, after adjusting for baseline outcome predictors our study showed that only hypotension and the presence of diffuse injury were independently predictive for in-hospital mortality. These data suggest that cardiac arrhythmias by itself are of less importance for survival in the acute phase after injury.

Another issue is whether these ECG abnormalities might be caused by primary cardiac dysfunction instead of reflecting a secondary effect of brain injury. After trauma, ECG abnormalities can reflect cardiac dysfunction, caused by thoracic injury, with potentially life-threatening complications that require intensive cardiac monitoring and treatment with vasoactive agents (16, 17). Since half of our patients had concomitant thoracic injury and we did not observe an association with specific ECG characteristics in patients with thoracic injury, cardiac injury as cause of ECG abnormalities was considered less likely. Besides, systemic effects could also influence the ECG pattern. Electrolyte disorders including abnormal potassium levels, which are associated with specific ECG abnormalities (36), were only present in one in five patients and were not associated with ECG abnormalities. This finding may be related to our policy of aggressive correction of potassium disturbances directly after admission. In ICU patients, supraventricular arrhythmias are well-recognized and have been reported as a results of increased catecholamine release (37). In trauma patients admitted to the ICU, atrial arrhythmias were reported in 7% (38), which is in accordance with our association of cardiac arrhythmias and a higher ISS. Conduction disorders were independently associated with presence of hypoxia, which is in line with prolongation of QTc-interval in healthy subjects during acute exposure to hypoxia (39). Since arrhythmias were more common in secondary deteriorated patients, combined with the absent relation with thoracic injury, our data suggest that ECG abnormalities more likely reflect the secondary effect of brain injury (i.e., cardiac mimicry) rather than primary cardiac dysfunction.

Despite the interesting findings of our study, several limitations have to be mentioned. First, our study has a retrospective design and therefore we did not obtain data on all ECGs of patients as assessment was driven by clinical care and not by study design. However, since no differences were present regarding patient characteristics between patients with and without available ECGs along with a considerable number of patients, we deem it likely to conclude that ECG abnormalities occur often in patients with severe TBI. Second, our findings are derived from patients with severe TBI who received ICP-monitoring in a single level-1 trauma center, so our results might not be comparable for all patients with severe TBI and for other centers, although our treatment is in accordance with international guideline-based management. Another limitation is hat in the current study primary cardiac injury as cause of ECG abnormalities was not determined systematically by echocardiography. So far two small prospective case-control studies reported no early major systemic myocardial depression to be present in patients with isolated severe TBI (15, 40). An objective of future studies could be to monitor systematically whether ECG abnormalities are related to primary cardiac ischemia by echocardiography and high-sensitive cardiac troponin T and compare these findings to non-head injured trauma patients.

In summary, in this study we aimed to evaluate the incidence and clinical significance of ECG abnormalities in the acute phase of severe traumatic brain injury. We observed a high incidence of ECG abnormalities without an attribution of location of traumatic lesions on CT-scan to ECG characteristics. Cardiac arrhythmias were associated with brain injury severity but were not an independent predictor for in-hospital mortality after traumatic brain injury. Therefore, our findings likely suggest that ECG abnormalities should be considered as cardiac mimicry representing the secondary effect of traumatic brain injury allowing for a more rationale use of neuroprotective measures.

## Data Availability Statement

The raw data supporting the conclusions of this article will be made available by the authors, without undue reservation.

## Ethics Statement

The studies involving human participants were reviewed and approved by the Medical Ethical committee of the University Medical Center Groningen (M11.096947). The ethics committee waived the requirement of written informed consent for participation.

## Author Contributions

JN, IH, and MN put together the study design and conceived the idea for the study. J-JL, JN, IH, MN, LK-K, BJ, SB drafted and revised the manuscript. J-JL, JN, IH, LK-K, MN acquired and interpreted the collected data. J-JL, JN and SB did the statistical analyses. All authors contributed to the article and approved the submitted version.

## Conflict of Interest

The authors declare that the research was conducted in the absence of any commercial or financial relationships that could be construed as a potential conflict of interest.
